# Phenotyping for drought adaptation in wheat using physiological traits

**DOI:** 10.3389/fphys.2012.00429

**Published:** 2012-11-16

**Authors:** Philippe Monneveux, Ruilian Jing, Satish C. Misra

**Affiliations:** ^1^International Potato CenterLima, Peru; ^2^Chinese Academy of Agricultural SciencesBeijing, China; ^3^Agharkar Research InstitutePune, India

**Keywords:** genetic resources, phenotyping traits, wheat, indirect selection, drought tolerance

## Abstract

Wheat (*Triticum* spp) is one of the first domesticated food crops. It represents the first source of calories (after rice) and an important source of proteins in developing countries. As a result of the Green Revolution, wheat yield sharply increased due to the use of improved varieties, irrigation, pesticides, and fertilizers. The rate of increase in world wheat production, however, slowed after 1980, except in China, India, and Pakistan. Being adapted to a wide range of moisture conditions, wheat is grown on more land area worldwide than any other crop, including in drought prone areas. In these marginal rain-fed environments where at least 60 m ha of wheat is grown, amount and distribution of rainfall are the predominant factors influencing yield variability. Intensive work has been carried out in the area of drought adaptation over the last decades. Breeding strategies for drought tolerance improvement include: definition of the target environment, choice and characterization of the testing environment, water stress management and characterization, and use of phenotyping traits with high heritability. The use of integrative traits, facilitated by the development and application of new technologies (thermal imaging, spectral reflectance, stable isotopes) is facilitating high throughput phenotyping and indirect selection, consequently favoring yield improvement in drought prone environments.

## General information

### Importance of wheat in the human diet

Wheat (*Triticum* spp) is one of the first domesticated food crops and, for 8000 years, has been the basic staple food of major civilizations of Europe, West Asia, and North Africa. Today, wheat continues to be an important food grain source for humans, and is a close third to rice and corn in total world production. Approximately two-thirds of the wheat produced is used for human food and about one-sixth for livestock feed. Industrial uses, seed requirements, and post-harvest losses account for the remainder. Wheat is used to produce a large variety of foods including many kinds of bread, cakes, noodles, crackers, breakfast foods, biscuits, and confectionary items. The protein content of wheat is in the range 12–16 percent and lipid content 1.5–2.0 percent.

World wheat production increased at a rate of 3.3 percent per year between 1949 and 1978. Increases at the start of this period were due to both an expansion of production area and increased yields. However, starting in the 1960s, yield increases came mainly from the use of improved varieties and a greatly expanded use of irrigation, pesticides, and fertilizers. The rate of increase in world wheat production slowed to 1.5 percent per year between 1982 and 1991, one exception being China, which maintained a rate of increase in production of 2.6 percent per year and became the world's largest wheat producer. Also, wheat production increased at nearly 3 percent per year in India and Pakistan during the same period.

Today, world wheat production is 626 million tons [Food and Agriculture Organization of the United Nations (FAO), 2007]. World leaders in order of production (all of the following figures are in million tons) are China (109), India (75.8), United States of America (55.8), Russian Federation (49.4), and France (32.8). Wheat is the dominant grain of world commerce. The five top exporters are USA (32.9), Canada (17.6), the European Community (16.5), Australia (14.7), and Argentina (9.6). The five top importers in 2007 were Brazil (6.6), Italy (6.2), Egypt (5.9), Japan (5.3), and Algeria (5.8).

### History of the crop, cultivated area, and yield performance under optimal conditions

Wheat is believed to have been domesticated in southwestern Asia (Gupta, 2004). Some of the earliest remains of the crop have been found in Syria, Jordan, and Turkey (Pasternak, 1998). Primitive relatives of present-day wheat have been discovered in some of the oldest excavations of the world in eastern Iraq, dating back 9000 years. Bread wheat is known to have been grown in the Nile valley from 5000 BC, and was later cultivated in other regions (e.g., the Indus and Euphrates valleys from 4000 BC, China from 2500 BC, and Europe from 2000 BC). It was introduced into the American continent around 1520.

Today, bread or common wheat (*T. aestivum* L) represents more than 90 percent of total wheat production. Several classes of bread wheat can be distinguished (hard red spring, hard red winter, soft red winter, hard white, and soft white) according to grain characteristics. Durum wheat or macaroni wheat (*T turgidum* subsp *durum* (Desf) Husnot) represents around 5 percent of global wheat production, i.e., 30 million tons. Durum wheat grain is hard, translucent, and large. The protein content can be as high as 18 percent. When durum is milled, the endosperm is ground into a granular product called “semolina,” used for premium pastas and breads. Due to its high level of tolerance to terminal drought, most durum wheat is grown in Mediterranean environments. The remaining part of the wheat growing area is distributed among the diploid species einkorn, the tetraploid species emmer, poulard, polish and timopheevi, and the hexaploid species spelt, club, compact and macha (Table 1).

**Table 1:** **Cultivated species (C) within the *Triticum* genus, and their wild relatives (W) (Source: van Slageren, 1994)**.

**Sections and species**	**Common name**		**Regions of cultivation**
**Section *Monococca* Flaksb**			
*Triticum monococcum L*			
Subsp.* monococcum*	Einkorn	C	Mountainous areas (France, Morocco, the former Yugoslavia, Turkey)
Subsp.* aegilopoides* (Link) Thell		W	
*Triticum urartu* Tumanian ex Gandilyan		W	
**Section *Dicoccoidea* Flaksb**			
*Triticum turgidum*			
Subsp.* turgidum*	Poulard	C	Mediterranean countries
Subsp. *carthlicum* (Nevski in Kom) Á Löve & D Löve		C	
Subsp. *dicoccum* (Schrank ex Schübler) Thell	Emmer	C	Yemen, India, Morocco, Spain, Albania, Turkey, Italy
Subsp. *durum* (Desf) Husnot	Durum	C	
Subsp. *paleocolchicum* (Menabde) Á Löve & D Löve		W	
Subsp. *polonicum* (L) Thell	Polish	C	Mediterranean countries
Subsp. *turanicum* (Jakubz) Á Löve & D Löve		W	
Subsp. *dicoccoides* (Körn ex Asch & Graebner) Thell		W	
*Triticum timopheevii* (Zhuk) Zhuk			
Subsp. *timopheevii*	Timopheevi	C	Georgia
Subsp. *armeniacum* (Jakubz) MacKey		W	
**Section *Triticum***			
*Triticum aestivum L*			
Subsp. *aestivum*	Bread	C	
Subsp. *compactum* (Host) MacKey	Compact	C	Alpine countries and Southern Europe
Subsp. *macha* (Dekapr & Menabde) MacKey		C	Caucasus area
Subsp. *spelta* (L) Thell	Spelt	C	Northern and Central Europe
Subsp. *sphaerococcum* (Percival) MacKey	Club	C	India
*Triticum zhukovskyi* Menabde & Ericzjan			

Being adapted to a wide range of moisture conditions from xerophytic to littoral, wheat is grown on more land area worldwide than any other crop. About three-quarters of the land area where wheat is grown receives between 375 and 875 mm of annual precipitation, but wheat can be grown in locations where precipitation ranges from 250 to 1750 mm. The optimum growing temperature is about 25°C, with minimum and maximum growth temperatures of 3–4°C and 30–32°C, respectively (Briggle and Curtis, 1987). Classification into spring or winter wheat traditionally refers to the season during which the crop is grown. For winter wheat, heading is delayed until the plant experiences a period of cold winter temperatures (0–5°C). It is planted in the autumn to germinate and develop into young plants that remain in the vegetative phase during the winter, and resume growth in early spring. This provides the advantage of using autumn moisture for germination and making effective use of early spring sunshine, warmth, and rainfall. Spring wheat, as the name implies, is usually planted in the spring and matures in late summer, but can be sown in autumn in countries that experience mild winters, such as in South Asia, North Africa, and the Middle East.

During the past 50 years, most of the yield progress in wheat has been due to the gradual replacement of traditional tall cultivars by dwarf and fertilizer-responsive varieties (Donmez et al., 2001; Brancourt-Hulmel et al., 2003; Jiang et al., 2003). Reducing height increased the proportion of carbon partitioned to grain and increased the harvest index (HI). It simultaneously reduced the risk of yield penalties caused by lodging. Most experiments analysing the effects of genetic improvement on yield found that, while selecting for higher-yielding cultivars, wheat breeders have consistently increased the number of grains per unit land area (Calderini et al., 1999). This is a consequence of a higher survival of floret primordia, the number of potential florets per spike remaining similar (Slafer and Andrade, 1993). It is likely to be due to pleiotropic effects on spike fertility of the two most commercially important gibberellic acid (GA)-insensitive dwarfing genes *Rht-B1b* and *Rht-D1b* (Flintham et al., 1997). Although there was an increase in grain number per m^2^, there was no reduction of grain weight, probably because photosynthetic capacity during grain filling together with pre-anthesis assimilate reserves exceed the demands of the growing wheat grains during post-anthesis (Borras et al., 2004).

Since the HI in most modern cultivars seems to be close to its biological maximum, i.e., 60 percent, further genetic gain in yield potential is expected to come from biomass increases (Shearman et al., 2005). Such increases have started to be reported in spring wheat (Reynolds et al., 1999) and winter bread wheat (Shearman et al., 2005). A biomass increase of about 10 percent has been reported in spring wheat, associated with the introduction of the long arm of chromosome 7D from the wheat wild relative *Lophopyrum elongatum* (Reynolds et al., 2001a; Monneveux et al., 2003). CIMMYT (Centro Internacional de Mejoramiento de Maíz y Trigo; the International Maize and Wheat Improvement Center) is exploring several approaches to exploit the excess photosynthetic capacity, like the multi-ovary characteristic which causes a single floret to set up to four kernels instead of just the usual one (Reynolds et al., 2005).

This requires, however, a good knowledge of the genetic and genomic resources available.

### Genetic and genomic resources

#### Genetic resources

Wheat belongs to the *Triticum* genus, family *Poaceae* Barnhart, subfamily *Pooideae*, tribe *Triticeae* Dumort, subtribe *Triticinae* Griseb (van Slageren, 1994). Kihara (1919) and Sax (1922) showed that, in the genus *Triticum*, there are three different genomes, each composed of seven chromosomes. Both genera *Aegilops* and *Triticum* (6 species and 17 subspecies) belong to a complex of wild and domesticated species of which the allopolyploid members evolved via hybrid speciation (Kimber and Sears, 1987), which have the same base chromosome number (*n* = 7), and can be divided into three ploidy levels (i.e., diploid 2n = 14, tetraploid 2n = 28, and hexaploid 2n = 42). Consequently, the available genepool of wheat is exceptionally wide (Zaharieva and Monneveux, 2006). As defined by Von Bothmer et al. (1992), the primary genepool consists of the cultivated and wild forms of a crop species. Gene transfer in the primary genepool is considered to be easy. The secondary genepool includes coenospecies from which gene transfer is possible but difficult, while the tertiary gene pool is composed of species from which gene transfer is very difficult.

Genetic resources have been categorized by Frankel (1975) as modern cultivars in current use, obsolete cultivars (i.e., the elite cultivars of the past, often found in the pedigrees of modern cultivars), landraces, wild relatives of the crop, genetic and cytogenetic stocks, and breeding lines. Today, CIMMYT's wheat germplasm bank holds more than 114,000 accessions, including parental and advanced breeding lines, cultivars and landraces, and more than 13,000 wild relatives from various regions of the world. Wheat genetic stocks involving translocation and substitution lines and produced by different institutions have been collected through a project supported by the Generation Challenge Programme (GCP), and are stored at CIMMYT.

#### Genomic resources

To address the need for general access to genetic maps, the International Triticeae Mapping Initiative (ITMI) was launched in 1989, to ensure that such maps would be available as a public good (Gustafson et al., 2004). Mapping using restriction fragment length polymorphisms (RFLPs) was conducted by scientists in several countries (McGuire and Qualset, 1997). As it became clear that resources for functional genomics, such as expressed sequence tags (ESTs), were the next critical need, ITMI created the International Triticeae EST Cooperative (ITEC). ITEC produced some 24,000 ESTs and mapped unigenes to chromosome bins defined by a set of deletion stocks. Then, a project on “The structure and function of the expressed portion of the wheat genomes” known as “wEST,” funded by the USA's National Science Foundation (NSF) Plant Genome Research Program along with support from the collaborating institutions, developed several activities such as complementary DNA (cDNA) library development, EST production, deletion stock characterization (Qi et al., 2003) and mapping, and coordination of individual chromosome maps. Project investigators and members of the international scientific community are free to use the ESTs for gene discovery and utilization. The ESTs are available from the Wheat Genomics Resource Repository—a collaboration between the United States Department of Agriculture (USDA) and the University of California, Davis, USA. It is now feasible to envisage the development of single nucleotide polymorphism (SNP) markers in wheat, due to the explosion in the availability of ESTs. The hexaploid nature of the wheat genome makes such analysis more complex than it would be in species with simple genomes. As an open international consortium of institutions (public and private), ITMI is now attempting to mine the contigs in a coordinated way, pooling information on validated SNPs and avoiding duplication of effort. Information on progress in this project can be found at: http://wheat.pw.usda.gov/ITMI/WheatSNP/

### Rationale for developing drought research in wheat

There has been a significant increase in the productivity of wheat due to the application of Green Revolution technology. This has resulted in a doubling and tripling of wheat production in many environments, most notably in irrigated areas. In these locations, the high-yielding semi-dwarf statured wheat cultivars continuously replaced the older tall types at a rate of 2 m ha year^−1^ in the 1980s (Byerlee and Moya, 1993). There is, however, a growing recognition that dissemination, application, and adoption of this technology have been slower in marginal environments, especially those affected by drought. Over the last three decades, many investigators have attempted to produce wheat cultivars adapted to these semi-arid environments with limited success in earlier years.

To examine the challenges facing wheat breeders more closely, Singh and Byerlee (1990) analysed wheat yield variability in 57 countries over 35 years. Yield variability was measured by calculating coefficients of variation of yield around linear climatic trends. The amount and distribution of rainfall was the predominant factor influencing yield variability. Countries in which half the wheat was sown in dryland conditions experienced twice as much variability as countries in which wheat was mostly grown under well-watered conditions. At least 60 m ha of wheat is grown in marginal rain-fed environments in developing countries. National average yields in these regions range from 0.8 to 1.5 t ha^−1^, which represents approximately 10–50 percent of their theoretical irrigated potential (Morris et al., 1991).

In recent years, breeders have been more successful in increasing the adaptation of wheat to dry environments. In developing countries, farmers have traditionally grown landrace cultivars that are well adapted to serious moisture stress conditions. However, these traditional cultivars generally give a poor yield in “good years” when rainfall is more plentiful. Modern cultivars now yield the same as the traditional cultivars in dry years as well as showing a better response to more favorable conditions of moisture and nutrient supply (Osmanzai et al., 1987). Due to their improved yield stability, these modern cultivars are increasingly grown in dry regions, with rates of adoption approaching those in irrigated and high rainfall areas.

Further progress in developing drought tolerant germplasm depends on the efficiency of breeding and phenotyping methodologies. Accurate drought phenotyping implies precise definition of the target environment, choice and characterization of the testing environment, and water stress management and characterization.

## Methodology

### Breeding strategy

Breeding work for drought-prone environments has been largely empirical to date, with grain yield being the primary trait for selection in wheat breeding programmes. However, most breeders select strongly for traits other than yield in the early segregating generations and do yield testing only at later stages, when a certain level of homozygosity has been achieved and large enough seed quantities are available. The decision to advance or reject a genotype is often complex and, in practical terms, breeders most often use a system of multiple cut-offs. In early generations, they select genotypes that, presumably, achieve the levels required for the primary traits evaluated in segregating populations (plant type, plant height, growth cycle, spike fertility, etc.).

When a breeding programme for drought adaptation is assisted by analytical selection, the conceptual model used considers yield under drought to be a function of: (1) yield potential; (2) flowering date (which indicates whether the crop will avoid drought stress); and (3) secondary traits that provide drought resistance. Physiological secondary traits can be used for the selection of parents to be included in the crossing block, as direct selection criteria for screening among a large number of genotypes (i.e., segregating populations) and/or when the amount of seed available is too small to carry out field trials with replications. Whereas intensive work is continuously being carried out by physiologists in the area of drought adaptation, few breeders routinely use physiological criteria in their mainstream breeding programmes. In the first place, the evaluation of some of the traits proposed by plant physiologists is time-consuming or expensive. This is not practical for application to the thousands of entries that comprise the segregating generations of breeding programmes. Then, the real value of a given trait may only be assessed by determining the genetic gain in segregating populations following selection, while many traits are not available in well adapted genotypes and their validation frequently requires the development of appropriate breeding material, which is again costly and time-consuming (Royo et al., 2005). Finally, selection in segregating populations requires screening at the plant level or between very small plots, thus hindering the use of traits that require large field plots for their assessment.

Gene-based markers generated from gene sequence data, i.e., “perfect markers” can be used to screen large numbers of entries for a particular trait improving the efficiency and effectiveness of conventional breeding. Gene-based markers are particularly useful for introgressing genes whose expression is highly affected by the environment, such as genes for useful physiological traits that cannot easily be screened (e.g., root architecture traits), as well as for gene pyramiding. The most common situations in which marker-assisted selection (MAS) confers an advantage are: (1) when accurate measurement of the phenotype is expensive or difficult; (2) when multiple genes conferring a similar phenotype are being combined; and (3) when there is a need for rapid removal of donor chromosome segments in a backcrossing programme (Nelson et al., 2004). Most important traits (yield, stress adaptation, etc.) are governed by multiple genes, each producing a relatively small individual effect. MAS for these “quantitative traits” is challenging because many quantitative trait loci (QTLs) identified in mapping population studies are cross-specific, subject to genotype-by-environment interaction (GEI) effects.

QTL estimation often spans several centimorgans, and hundreds of genes underlie a region of this size. The size of such a region can be reduced through a number of approaches, such as the use of high resolution crosses, or the development of near-isogenic lines (NILs) for small chromosomal segments across the putative QTL region (Nelson et al., 2004). Linkage disequilibrium (LD) mapping offers another alternative, exploiting the long history of recombination, and rich allelic diversity in germplasm collections (Remington et al., 2001; Buckler and Thornsberry, 2002). Genome sequencing for various crops would improve the quality of molecular markers used for MAS by helping breeders to target the gene of interest, rather than a nearby sequence (Dubcovsky, 2004). Continuing efforts to sequence expressed genes will provide data for SNP markers for individual alleles, making MAS more cost-efficient (Dubcovsky, 2004).

For MAS to be useful, proper phenotyping is required and the evaluation of yield and relevant physiological traits should be done in conditions similar those of the target environment. An ecophysiological understanding of the traits in question and of how to measure them is crucial (Araus et al., 2003a,b; Slafer, 2003).

### Trial planning

#### Definition of the target environment

Rainfall distribution patterns and evaporative demand over the crop cycle vary considerably among locations and years. The different sets of climatic conditions under which wheat is cultivated are characterized by breeders as “wheat mega-environments” (wheat MEs). ME delineation is based on water availability, soil type, temperature regime, production system, and associated biotic and abiotic stresses. Consumer preferences for grain color and industrial and end-use quality are also considered. CIMMYT has defined 12 MEs (Table 2): six focus on spring wheat production areas, three on facultative wheat areas, and three on true winter wheat areas (Rajaram et al., 1995). According to the ME classification, drought environments mainly correspond to ME4. Within ME4, three distinct patterns can be distinguished: post-anthesis water stress (ME4A); pre-anthesis water stress (ME4B); and residual moisture stress (ME4C). In the first scenario, ME4A, evapotranspiration exceeds average precipitation after anthesis, causing an increasing water deficit over the grain-filling period. Conversely, in ME4B, water deficit occurs mainly before anthesis. In ME4C, there is no significant rainfall, and evaporation is always in excess of precipitation during the growing season. Consequently, the crop must survive using the water stored in the soil profile from the summer rainfall. Wheat can also face drought situations in other MEs, such as ME6B, ME9, and ME12. In all drought situations, wheat may also experience additional stresses such as heat and cold stress, soil micro-element deficiency, or toxicity, and a range of biotic stresses. For example, late frosts frequently occur in ME4A, while high temperature stress occurs in ME5A. In ME4B, resistances to leaf and stem rust, *Septoria* spp and *Fusarium* spp, and pre-harvest sprouting are highly necessary.

**Table 2:** **The main wheat mega-environments (Source: Rajaram et al., 1995)**.

**ME^*a*^**	**Moisture regime**	**Temperature**	**Wheat type**	**Area (%)**	**Production (10^6^ tons)**
ME1 IR	IR	Temperate	Spring	36.1	83
ME2 HR	HR (>500 mm)	Temperate	Spring	8.5	25
ME3 AS	HR (>500 mm); AS	Temperate	Spring	1.9	3
ME4 SA	LR (<500 mm)	Temperate/hot	Spring	14.6	20
ME5 TE	IR, HR	Hot	Spring	7.1	12
ME6 HL	SA	Temperate	Spring	6.2	13
ME7 IR	IR	Cool	Facultative	−	−
ME8 HR	HR	Cool	Facultative	10.0	23
ME9 SA	SA	Cool	Facultative	−	−
ME10 IR	IR	Cold	Winter	−	−
ME11 HR	HR	Cold	Winter	15.0	30
ME12 SA	SA	Cold	Winter	−	−

a ME, Mega-environment; where: IR, irrigated; HR, high rainfall; AS, acid soil; SA, semi-arid; TE, tropical environment; HL, high latitude.

#### Choice and characterization of the testing environment

The choice of the selection environment directly determines the potential genetic gains in the target environment. Ideally, the selection environment should mimic the target environment in all aspects: water distribution, profiles and potential evapotranspiration rates, and physical and chemical soil properties. Deviations may result in significant GEI between target and selection environments, and genetic gains achieved in the selection environment may not be expressed in the target environment. Geographic information system (GIS) tools can help considerably in describing the relationships between target and selection environments and establishing “homology maps.”

The crop facing water deficit simultaneously experiences a number of additional stress factors (e.g., micronutrient deficiency, soil compaction, salinity, nematodes, and fungal pathogens) that exacerbate drought stress. Such factors are hard to control and are generally not considered in field experiments. Hence, efforts should be made to remove all other constraints except drought. Soil surveys may allow the identification of selection sites or fields that avoid confounding factors. In some cases, these surveys may enable sites to be chosen where the selection pressure for these stress factors would permit the selection of genotypes targeted for regions where these stresses interact with drought. They could also identify the within-site distribution of e.g., nematodes (Nicol and Ortiz-Monasterio, 2004) or zinc deficiency (Ekiz et al., 1998). Field trials are conducted on land that may be quite variable in terms of topography, soil fertility, and soil structure. Spatial variability in the field affects the detection of treatment differences in agricultural experiments by inflating the estimated experimental error variance. In order to account for such variation and to reduce experimental error, adapted trial designs must be applied, like the augmented designs proposed by Federer (2005).

### Water stress management and characterization

Target environments can also be mimicked if water is controlled by imposing a water regime by gravity or, better, by drip irrigation. Water stress management (timing, intensity, uniformity) and characterization (soil, plant measurements) are essential issues in drought phenotyping.

Moisture availability can itself be a complicating factor when comparing genotypes in field experiments. Although plots growing the different genotypes may receive the same quantity of water, the genotypes can vary in their water use and/or access to underground water, thereby confounding measurements associated with plant water relations. Study of water profiles (either experimentally or by using simulation models) can provide very useful information. Trait evaluation should preferably be carried out under field conditions, avoiding experimental situations (growth chambers, greenhouses, pots) that differ significantly from the agricultural growing environment. The ability to access water deep in the soil profile, which is an important drought-adaptive mechanism, is eliminated as a variable in pot conditions. Furthermore, the relative humidity of the air, which has an important influence on stomatal conductance (Ben Haj Salah and Tardieu, 1997), is extremely difficult to simulate in controlled environments.

When possible, drought tolerance evaluation should be done out-of-season, under irrigated conditions. This option allows better management of water stress but needs a dry season sufficiently long to cover the whole growth cycle. The photoperiod and temperature should not differ too much from the growing season, as is the case in the dry tropics, to avoid genotype-by-season interactions and allow results obtained from the out-of-season experiments to be extrapolated to the growing season conditions.

### Plant water strategy

#### Survival and drought escape

In the case of drought, some traits proposed by stress physiologists appear to be associated with crop survival. For example, comparison of old and new varieties has shown that, under drought, older varieties over-produce tillers many of which fail to set grain, while modern drought tolerant lines produce fewer tillers the majority of which survive (Loss and Siddique, 1994). In most circumstances, however, the main effect of drought is to reduce grain yield without killing the plant.

If the pattern of water deficit is predictable in a given region, selection for a flowering date that does not coincide with the period of water deficit is a very effective way of improving drought adaptation (Araus et al., 2002). The limitations of this approach are that very early varieties may suffer yield penalties in good seasons, while late-in-season freezing episodes may affect spike fertility. In such cases, breeding for higher yield potential plus traits conferring stress avoidance (i.e., to avoid cell dehydration) may generally be effective (Araus et al., 2003a,b).

### Phenotyping traits

#### Requirements

Most of the traits currently mentioned in the literature associated with drought adaptation in wheat are shown in Table 3. However, the potential value of each trait needs to be considered with respect to the type of drought environment in which a cultivar is to be adapted. Secondary traits may be particularly suited to improving the selection response for stress conditions if they avoid any confounding effects of stress timing on yield (e.g., drought and flowering dates), and allow the selection to be focused on a specific type of drought. For a secondary trait to be useful in a breeding programme, it has also to comply with several requirements (Edmeades et al., 1997). Thus, a secondary trait should ideally be: (1) genetically associated with grain yield under drought; (2) genetically variable; (3) highly heritable; (4) easy, inexpensive and fast to observe or measure; (5) non-destructive; (6) stable over the measurement period; and (7) not associated with yield loss under unstressed conditions.

**Table 3:** **Main secondary traits that can be used to improve drought tolerance in wheat, associated mechanisms, references, ease of use, and target mega-environment of application (Adapted from Reynolds et al., 2001b)**.

**Secondary trait**	**Associated with**	**Methodology (References)**	**Ease of use**	**Target environment**
Large seed size	Emergence, early ground cover, and initial biomass	Mian and Nafziger, 1994	+++	ME4A
Long coleoptiles	Emergence from deep sowing	Radford, 1987	+++	ME4C
Early ground cover (visual)	Decrease of evaporation and increase of radiation-use efficiency (RUE)	Hafid et al., 1998; Richards, 1996	+++	ME4A
Specific leaf dry weight	Thinner, wider leaves, early ground cover	Merah et al., 2001a	++	ME4A
Growth habit (visual)	Lower soil evaporation and higher RUE	Richards et al., 2002	+++	ME4A
Tiller survival	Survival and recovery	Loss and Siddique, 1994	++	Severe stress
Long and thick stem internodes	Storage of carbon products	Loss and Siddique, 1994	+++	ME4A
Vegetation indices (normalized difference vegetation index; NDVI)	Green biomass	Royo et al., 2003	+	
Earliness	Drought escape	Blum, 1988; Monneveux et al., 2005	+++	ME4A and ME4C
Number of grain per spike around	Spike sterility	Hafsi et al., 2006	++	Drought flowering
Stomatal conductance	Extraction of water from soil	Farquhar and Sharkey, 1982	+	
Canopy temperature depression	Stomatal conductance, extraction of water from soil	Reynolds et al., 2000	++	
Carbon isotope discrimination	Stomatal conductance, extraction of water from soil	Monneveux et al., 2005	++	
Ash content	Stomatal conductance, extraction of water from soil	Misra et al., 2006	++	
Spike photosynthetic capacity	Grain filling	Evans et al., 1972	+	ME4A, hot
Leaf color (visual, SPAD)	Delayed senescence, maintenance of photosynthesis	Araus et al., 1997	+++	
Leaf waxiness	Lower transpiration rate and reduced photo-inhibition	Richards, 1996	+++	Severe stress
Leaf pubescence	Lower transpiration rate and reduced photo-inhibition	Richards, 1996	+++	Severe stress
Leaf thickness and posture	Lower transpiration rate and reduced photo-inhibition	Reynolds et al., 2000	+++	Severe stress
Leaf rolling	Lower transpiration rate and reduced photo-inhibition	Reynolds et al., 2001b	+++	Severe stress
Glume pubescence	Lower transpiration rate and reduced photo-inhibition	Trethowan et al., 1998	+++	
Delayed senescence	Higher RUE	Hafsi et al., 2006	++	
Fructanes in stem	Storage of carbon products	Rawson and Evans, 1971	++	ME4A
Solute concentration in cells	Osmotic adjustment (OA)	Morgan and Condon, 1986	+	
Accumulation of ABA	Reduced stomatal conductance and cell division	Innes et al., 1984	+	Severe stress

Wheat faces different drought scenarios worldwide; consequently, the physiological traits that confer drought resistance in specific environments may be very distinct. The combination of yield data with data relating to secondary traits in multi-site field experiments ranging from well-watered to high stress levels may be useful at this stage by providing some light on GEI of traits related to drought tolerance. This is particularly the case when the heritability of the secondary traits is higher than that of yield, and the genetic correlation of these traits with yield in the target environment is high. Secondary traits can be classified according to their relationship to pre-anthesis growth, access to water, water-use efficiency (WUE), and photoprotection.

#### Traits related to pre-anthesis growth

***Crop establishment.*** Vigorous crop establishment is agronomically desirable because it helps to shade the soil and suppress weeds that compete for water. It also improves radiation interception by the crop at the early stages of growth. Rapid ground cover can be achieved by breeding for: (1) large seed and embryo size which may help to achieve early vigor (Aparicio et al., 2002); and (2) thinner, wider leaves (i.e., with a relatively low specific leaf weight) and a more prostrate growth habit which help to increase ground cover, thus conserving soil moisture and potentially increasing radiation-use efficiency (RUE; Richards, 1996). These traits are especially important in Mediterranean types of drought environment (ME4A), where up to 40 percent of available water may be lost by evaporation directly from the soil (Loss and Siddique, 1994). In ME4A, the potential for vigorous growth prior to heading also provides the opportunity to take advantage of relatively good growing temperatures and moisture availability earlier in the cycle.

***Total biomass.*** Evaluation of total biomass is only feasible in practice through indirect methods, e.g., using spectroradiometers to measure the spectra of light reflected by the canopy (Aparicio et al., 2000, 2002; Royo et al., 2003). Field spectroradiometers able to measure the spectrum of light reflected by the canopy have been expensive in the past. However, the situation is now changing with the availability of simple, less expensive, and easy-to-handle spectroradiometers such as the GreenSeeker^1^. Designed initially for nitrogen management, this has become a potentially very useful instrument in breeding. It gives the basic spectroradiometric indices of green biomass, such as the normalized difference vegetation index (NDVI), which is the most useful for routine breeding purposes. Moreover, as the GreenSeeker includes its own radiation source, it may be used independently of atmospheric conditions and deployed on both sunny and cloudy days. Alternative techniques such as the use of an affordable conventional digital camera may provide complementary information, such as the portion of the soil occupied by green biomass (Casadesús et al., 2005). Digital pictures may also provide information that is not currently acquired through spectral reflectance measurements, such as the degree of soil covered by the crop, the percentage of yellow leaves, or even yield components such as the number of spikes per unit land area (Casadesús et al., 2005).

***Remobilization of stored assimilates.*** Stored assimilates can be remobilized during grain filling to supplement assimilates generated in the drier post-anthesis period. Stored fructans contribute substantially to grain filling, especially when canopy photosynthesis is inhibited by post-anthesis drought (Blum, 1998). Traits that may also contribute to remobilization during grain filling in these conditions include long and thick stem internodes, perhaps with extra storage tissue in the form of solid stems. In studies where crosses were made between lines contrasting in the solid-stem trait, the solid-stem progeny contained more soluble carbohydrate per unit of stem length, although total stem carbohydrate was unaffected due to the stems being narrower and shorter (Ford et al., 1979). Conversely, where the crop grows exclusively on stored soil moisture, long coleoptiles are desirable to avoid extremely hot soil surface temperatures and rapid soil drying. Moreover, longer coleoptiles improve seedling emergence with deep sowing (Radford, 1987), improving early biomass accumulation (Rebetzke et al., 2005). Long coleoptiles are also useful in dryland Mediterranean environments, helping to avoid a false start in early planted crops.

#### Traits relating to access to water

***Root characteristics.*** A root system that can extract whatever water is available in the soil profile is clearly drought-adaptive (Hurd, 1968), but this ability is difficult to measure directly. Traits that are indicative of the water status of a plant, especially when measured during periods of peak stress, are useful indicators of the plant's capacity to match evaporative demand by exploring and extracting soil water. Instantaneous measurement of traits affected by the water relations of the plant, such as stomatal conductance (*g*_*s*_) and canopy temperature depression (CTD) can give indications of water extraction patterns. The role of abscisic acid (ABA) accumulation in stomatal regulation under drought has been demonstrated (Innes et al., 1984). It also appears to preadapt plants to stress by reducing rates of cell division, reducing organ size, and increasing the rate of development. However, high ABA can also result in sterility problems since high ABA levels may abort developing florets.

***Canopy temperature depression.*** Among the traits relating to access to water, by far the easiest to measure in the field is CTD, which shows good correlations with other water relations parameters (Blum et al., 1982), as well as with performance under drought of random sister lines (Reynolds et al., 2000). Canopy temperature can provide information on transpiration as the main contributor to reduced leaf temperature. Although canopy temperature may seem very easy to measure, in practice there are methodological problems, particularly in Mediterranean drought environments. This is mainly found when there is variation in the air temperature with wind or cloudiness (Araus et al., 2002; Royo et al., 2002), or where there is not a homogeneous canopy. In fact, screening by canopy temperature measurements under drought stress can be done only during the vegetative growth stage after full ground cover has been attained, before inflorescence emergence, at high vapour-pressure deficits in recently irrigated crops, and without the presence of wind or clouds (Royo et al., 2005).

So far, studies have only been accomplished in recombinant inbred lines (RILs). CTD showed a significant association with yield under drought when measured pre-anthesis, suggesting an advantage from higher pre-anthesis growth rates. CTD also showed some association with final yield when measured during grain filling. Because a major role of transpiration is leaf cooling, canopy temperature, and its reduction relative to ambient air temperature are an indication of how much transpiration cools the leaves under a demanding environmental load. Higher transpiration means colder leaves and higher stomatal conductance, both aspects favoring net photosynthesis and crop duration. A relatively lower canopy temperature in drought-stressed crops indicates a relatively greater capacity for taking up soil moisture or for maintaining a better plant water status. Thus, higher transpiration is a positive trait when selecting for higher yield potential or better adaptation to moderate drought stress.

***Osmotic adjustment.*** Solute concentration in the cell is intimately tied to plant water status and, under drought, osmotic adjustment (OA) may facilitate critical growth functions such as root growth, and also meiosis and pollen development, thereby mitigating some of the most detrimental effects of plant water deficit. Genetic variation in OA is well-established in wheat (Rekika et al., 1998). A number of experiments have shown that wheat lines selected for high OA in response to the lowering of leaf water potential have higher grain yields in field experiments. In a study by Morgan and Condon (1986), high OA was strongly associated with greater soil water extraction. Nevertheless, the role of OA on yield still remains controversial (Serraj and Sinclair, 2002). It will help to maintain leaf metabolism and root growth at relatively low leaf water potentials by maintaining turgor pressure in cells. However, OA is difficult to measure in large samples under field conditions. Some research suggests that the trait can be assayed relatively easily by measuring the coleoptile growth rate of seedlings in a polyethylene glycol (PEG) solution (Morgan, 1988).

***Carbon isotope discrimination.*** Carbon isotope discrimination (Δ^13^C), despite being a very promising trait, is probably less widely accepted because of the cost of its determination. In recent years, Commonwealth Scientific and Industrial Research Organisation (CSIRO, Australia) Plant Industry has released the first two commercial wheat varieties selected for high transpiration efficiency using Δ^13^C (“Drysdale” in 2002, and “Rees” in 2003). These varieties are cultivated under rain-fed conditions and rely solely upon the precipitation accumulated prior to planting. They have been selected based on their low Δ^13^C (and thus high transpiration efficiency), fitting with what has been postulated with regard to this trait. However, for Mediterranean environments, Δ^13^C (particularly when measured in mature grains) is frequently positively correlated with grain yield (Araus et al., 1998; Villegas et al., 2000; Merah et al., 2001b; Condon et al., 2004; Monneveux et al., 2005). One of the reasons for this positive relationship is that a genotype exhibiting higher Δ^13^C is probably able to maintain a better water status (Condon et al., 2004). Given the relatively high costs associated with carbon isotopic analysis (about €10 per sample), several surrogate approaches which are much cheaper, faster, and easier to handle have been proposed. The option most studied has been to use the mineral or ash content of leaves (Masle et al., 1992; Mayland et al., 1993; Araus et al., 1998; Merah et al., 1999) or grains (Febrero et al., 1994; Voltas et al., 1998, Monneveux et al., 2005; Misra et al., 2006). Another promising alternative relies on the estimation of Δ^13^C through the near-infrared spectroscopy (NIRS) technique (Clark et al., 1995; Ferrio et al., 2001), which carries with it the further advantage of being non-destructive.

#### Traits relating to water-use efficiency

Measurement of carbon isotope discrimination or ash content of grain or other tissues can be used to estimate the WUE of the crop, since their signals are based on the integration of plant water status over a period of time (Condon et al., 1993). However, these data must be interpreted with care. Although most field studies have shown that better performance of wheat cultivars under Mediterranean drought conditions is associated with lower WUE (Condon et al., 1993), studies in Australia (Rebetzke et al., 2002) indicated an advantage for high WUE genotypes under conditions where crops survive exclusively on stored soil moisture.

Spikes have higher WUE than leaves, and have been shown to contribute up to 40 percent of total carbon fixation under moisture stress (Evans et al., 1972). Awns contribute substantially to spike photosynthesis and longer awns are a possible selection criterion. While gas exchange measurement of spikes is time consuming and difficult to standardize, chlorophyll fluorescence should be considered as a more rapid means of screening for spike photosynthetic capacity under stress (Horton, pers. communication).

Genes that affect a greater relative partitioning of assimilates to the sink, resulting in a higher HI, would be expected to improve yield under drought, not being associated with the water cost of generating additional biomass. Plant height is usually negatively related with HI. However, there is a minimum height below which limitation on yield becomes evident (Slafer et al., 2005). Under extreme drought stress where most of the canopy may have senesced, spike photosynthesis can play a major role in grain filling, because of high WUE of the spike due to the fact than they can refix respiratory carbon (Bort et al., 1996). Moreover, they are able to maintain a better water status than leaves, through a higher OA and a more xeromorphic structure (Tambussi et al., 2005). This stay-green spike trait is currently being introgressed by CIMMYT into elite drought-tolerant backgrounds to see if it can be combined with yield responsiveness, such that the trait is facultative, responding only in drier years. Changes in leaf color can reflect a variation in partitioning of assimilates to the sink. Stress may accelerate the senescence of leaves. The stay-green trait may indicate the presence of drought avoidance mechanisms, but probably does not contribute to yield per se if there is no water left in the soil profile by the end of the cycle to support leaf gas exchange. It may be detrimental if it indicates lack of ability to remobilize stem reserves (Blum, 1998).

To check for delayed senescence of leaves, particularly flag leaves, portable chlorophyll meters such as the Minolta SPAD^2^ are extensively used, due to their speed and ease of use. Delayed senescence of leaves has been proposed as a secondary trait for performance under drought by several authors (Araus et al., 1997; Rharrabti et al., 2001). However, the relationship between delayed senescence and yield has been found by other authors to be unstable and highly dependent on drought intensity (Hafsi et al., 2006). In addition, the cost of a portable chlorophyll meter makes this device unaffordable for many breeding programmes in developing countries.

#### Traits relating to photoprotection

Decreased stomatal conductance in response to drought leads to warmer leaf temperatures and insufficient CO_2_ to dissipate incident radiation, both of which increase the accumulation of harmful oxygen radicals and photo-inhibitory damage. Photo-inhibition can be modified by some leaf adaptive traits such as waxiness, pubescence, rolling, thickness, or posture (Richards, 1996). These traits decrease the radiation load to the leaf surface. Benefits include a lower evapotranspiration rate and reduced risk of irreversible photo-inhibition. However, they may also be associated with reduce RUE, which would reduce yield under more favorable conditions.

The effects of photo-inhibition can be alleviated by antioxidants such as superoxide dismutase (SOD) and ascorbate peroxidise, which have been shown to increase in quantity in response to drought stress (Mittler and Zilinskas, 1994). Thermal dissipation through the xanthophyll cycle is another protective mechanism that can dissipate as much as 75 percent of absorbed light energy (Niyogi, 1999). In a study comparing a drought-adapted barley landrace with a modern cultivar, the former displayed two mechanisms of photoprotection: (1) rapid xanthophyll cycling; and (2) up to 50 percent less leaf chlorophyll, resulting in a passive reduction of light absorbance (Havaux and Tardy, 1999).

#### Application in breeding

While many traits have been studied for their use in breeding for drought resistance, there is a general consensus among breeders that only a few of them can be recommended for use in practical breeding programmes at this time (Table 3). For example, CIMMYT (Reynolds et al., 2001a) recommend the use of flowering and maturity dates, spike fertility, changes in green biomass (e.g., leaf death score), and canopy temperature. In practical terms, these traits seem valuable when breeding for higher yield potential and adaptation to some degree of stress. Development of new equipment like spectroradiometers will facilitate future measurement of new physiological traits in the field. Most other traits cannot yet be recommended as part of an ongoing breeding programme, particularly those that are expensive or difficult to measure. However, some such as Δ^13^C can be used for the selection of parents (Misra et al., 2006; Xu et al., 2007). Thermal imaging and color imaging techniques are expected to greatly facilitate large scale evaluations in the next future (Cabrera-Bosquet et al., 2012).

## Conclusions

Many drought-adaptive traits have been investigated in wheat. However, association of these traits with genetic gains for yield under drought has been poorly tested and documented. Most difficulties encountered in the identification of accurate drought tolerance traits are due to the fact that wheat is cultivated under very different climatic conditions and faces very different drought scenarios worldwide.

While some single traits have benefited from tremendous research efforts and have generated considerable debate in the literature (e.g., OA, ABA), relatively little emphasis has been placed on research that can be extrapolated and used directly to crop genetic improvement in target environments.

Most drought physiology research in wheat has been conducted in controlled environments and has been poorly integrated into breeding programmes. Multidisciplinary approaches involving physiologist, breeders, genebank managers, and biotechnologists are still scarce, holding back the exploitation of genetic diversity and the use of MAS for drought tolerance improvement.

Despite the tremendous potential offered by access to genetic resources from related species, and well-documented success in using them (e.g., 1B/1R translocation, synthetic wheats), wild *Triticeae* have been poorly exploited until now to improve drought tolerance in wheat.

### Conflict of interest statement

The authors declare that the research was conducted in the absence of any commercial or financial relationships that could be construed as a potential conflict of interest.

## Footnotes

^1^http://www.ntechindustries.com/

^2^http://www.specmeters.com/Chlorophyll_Meters/Minolta_SPAD_502_Meter.html
